# Impacts of p97 on Proteome Changes in Human Cells during Coronaviral Replication

**DOI:** 10.3390/cells10112953

**Published:** 2021-10-29

**Authors:** Kai-Wen Cheng, Shan Li, Feng Wang, Nallely M. Ruiz-Lopez, Nadia Houerbi, Tsui-Fen Chou

**Affiliations:** 1 Division of Biology and Biological Engineering, California Institute of Technology, Pasadena, CA 91125, USA; lflshan@caltech.edu (S.L.); fengwang@caltech.edu (F.W.); naynayr88@gmail.com (N.M.R.-L.); nhouerbi@colgate.edu (N.H.); 2 Proteome Exploration Laboratory, Beckman Institute, California Institute of Technology, Pasadena, CA 91125, USA

**Keywords:** coronavirus, p97, ATPase, proteomics, inhibitor

## Abstract

Human coronavirus (HCoV) similar to other viruses rely on host cell machinery for both replication and to spread. The p97/VCP ATPase is associated with diverse pathways that may favor HCoV replication. In this study, we assessed the role of p97 and associated host responses in human lung cell line H1299 after HCoV-229E or HCoV-OC43 infection. Inhibition of p97 function by small molecule inhibitors shows antiviral activity, particularly at early stages of the virus life cycle, during virus uncoating and viral RNA replication. Importantly, p97 activity inhibition protects human cells against HCoV-induced cytopathic effects. The p97 knockdown also inhibits viral production in infected cells. Unbiased quantitative proteomics analyses reveal that HCoV-OC43 infection resulted in proteome changes enriched in cellular senescence and DNA repair during virus replication. Further analysis of protein changes between infected cells with control and p97 shRNA identifies cell cycle pathways for both HCoV-229E and HCoV-OC43 infection. Together, our data indicate a role for the essential host protein p97 in supporting HCoV replication, suggesting that p97 is a therapeutic target to treat HCoV infection.

## 1. Introduction

Coronaviruses (CoVs) are a family of enveloped positive-sense single-stranded RNA viruses, that are linked to respiratory and enteric disease [1]. The CoVs are classified under the subfamily *Coronovirinae* within the family *Coronaviridae* and the order *Nidovirales*. Based on genome sequences and phylogenetic relationships, members of the *Coronavirinae* subfamily are further divided into four groups, *Alphacoronaviruses*, *Betacoronaviruses*, *Gammacoronaviruses*, and *Deltacoronaviruses* [1]. To date, seven human CoVs (HCoVs) have been identified. The HCoV-229E and HCoV-NL63 belong to the *Alphacoronaviruses*, whereas the others belong to three *Betacoronavirus* lineages. The HCoV-OC43 and HCoV-HKU1 belong to lineage A, and the SARS-CoV and SARS-CoV-2 belong to lineage B while MERS-CoV belongs to lineage C [2]. Unlike the four common HCoVs (229E, OC43, NL63, and HKU1), which generally cause mild to moderate upper-respiratory tract infections similar to the common cold, SARS-CoV, SARS-CoV-2, and MERS-CoV cause severe, acute respiratory pathologies [3]. The SARS-CoV-2 is highly transmissible and pathogenic and causes coronavirus disease 2019 (COVID-19) [3]. There is currently only one drug approved by the U.S. Food and Drug Administration (FDA) for the treatment of hospitalized COVID-19 patients [4]. This drug, remdesivir, is a nucleotide analogue prodrug that perturbs viral replication by inhibiting the RNA-dependent RNA polymerase (RdRP) [5]. Other current therapies are primarily supportive. As such, novel therapeutic agents that can inhibit infection and virus replication are urgently needed.

Generally, CoV infection is initiated by binding of the viral spike (S) protein to its cellular receptor. These receptors are human aminopeptidase N (APN) for HCoV-229E [6], angiotensin-converting enzyme 2 (ACE2) for HCoV-NL63, SARS-CoV, and SARS-CoV-2 [7,8,9], and dipeptidyl peptidase 4 (DPP4) for MERS-CoV [10]. Host proteases cleave the S protein upon receptor binding, triggering internalization of the virus into the host cell [11]. Internalization can either occur through direct fusion of the viral envelope with the plasma membrane or within endosomes, depending on the virus strain and host cell type. This step leads to the release of viral nucleocapsids into the cytoplasm of host cells [12]. After uncoating, the viral genome is immediately translated into two coterminal polyproteins (pp), pp1a and pp1ab, that can be further cleaved by viral proteases into non-structural proteins (NSPs). These NSPs form the viral replication/transcription complex (RTC), required for RNA replication and transcription [1]. Ultimately, viral structural proteins and the RNA genome are assembled in the ER-Golgi intermediate compartment (ERGIC) to form mature virions, which are transported to the cell surface in vesicles and released via exocytosis [1]. As viruses rely on host cell machinery for both replication and to spread, blocking key host factors required for these processes may provide a broad-spectrum therapeutic approach to HCoV infection.

The AAA+ ATPase p97, also known as a valosin-containing protein (VCP), is a conserved and abundant eukaryotic protein that uses the energy of ATP hydrolysis to promote conformational changes in substrate proteins [13]. The p97 binds ubiquitylated proteins and dissociates them from macromolecular complexes. In some cases, it facilitates the downstream degradation of substrates through the ubiquitin proteasome system (UPS) [13]. It is important to note that p97 cooperates with various cofactors and adaptors, and is essential in diverse cellular functions. These cellular processes include ER associated degradation (ERAD) [14], cell cycle regulation [15], autophagy, and the lysosomal function [16]. Interestingly, these cellular processes are highly related to the host cell machinery hijacked by viruses to promote viral replication in infected cells [17,18,19,20]. Given that p97 is a key factor in various pathways that may favor viral replication in host cells, p97 inhibition might interfere with HCoV replication, making this a plausible therapeutic target.

To explore the role of p97 in host responses during HCoV replication, we firstly examined whether p97 inhibition interferes with HCoV replication and the resulting cytopathic effect (CPE). We then elucidated the impact of p97 on early stages of the viral life cycle using p97 inhibitors. We also generated a p97-knockdown cell line and used to determine the effects of p97 depletion on HCoV-229E or HCoV-OC43 replication. Finally, we employed unbiased quantitative proteomics to analyze changes in host cellular processes after HCoV infection in the presence or absence of p97. Together, our data indicate the potential host pathways that p97 is involved in supporting virus replication, suggesting that development of p97 inhibitors is a promising avenue for treating HCoV infection.

## 2. Materials and Methods

### 2.1. Cell Lines

The 293T, A549, H1299, MRC-5, and HCT-8 cells were purchased from ATCC. Cells were cultured in DMEM media (MilliporeSigma, St. Louis, MO, USA) or RPMI 1640 media (Corning, Manassa, VA, USA) supplemented with 10% fetal bovine serum (FBS, Atlanta Biologicals), 100 μg/mL streptomycin, and 100 U/mL of penicillin (Lonza, Walkersville, MD, USA) and maintained at 37 °C with 5% CO_2_.

### 2.2. Chemical Inhibitors

The p97 inhibitors, CB-5083 (MedKoo Biosciences, Inc., Morrisville, NC, USA) and NMS-873 (Xcess Biosciences Inc., San Diego, CA, USA) were dissolved in DMSO at stock concentration of 10 mM.

### 2.3. Generation of p97-Knockdown Ccell Line

Stable H1299 cell lines expressing doxycycline (Dox)-inducible shRNA against a control sequence or p97 were generated using the TripZ lenti-viral shRNA system (Thermo Fisher Scientific, Carlsbad, CA, USA) as described previously [21]. The targeted sequence for p97 is 5′-AAACAGCCATTCTCAAACAGAA-3′. The nontargeting control shRNA comes directly from Thermo Fisher Scientific. The control or p97 shRNA plasmid and lentivirus packaging plasmids (pHDM-G, CAG4-RTR2, and CAGGHIVgpco) were transiently co-transfected into 293T cells with BioT reagent (Bioland Scientific LLC, Paramount, CA, USA). Supernatant containing the lentivirus was collected and supplemented with polybrene (8 μg/mL) to transduce H1299 cells.

### 2.4. HCoV Stock Production and Viral Titer Titration

The HCoV-229E strain (ATCC, VR-740) and HCoV-OC43 strain (ATCC, VR-1558) were purchased from ATCC, passaged once through MRC-5 cells (ATCC, CCL-171) and HCT-8 cells (ATCC, CCL-244), respectively, and were amplified in H1299 (ATCC, CRL-5803) or A549 cells (ATCC, CCL-185). Between 80 and 90% of the confluent cells were infected with HCoV-229E and HcoV-OC43 in a minimal volume of serum-free media for 2 h at 35 °C and 33 °C, respectively. Infected cells were incubated in media containing 2% FBS for 4 to 5 days. Culture supernatants were harvested when the CPE was observed, centrifuged at 4000 rpm for 5 min, passed through a 0.45 μm filter, and aliquoted for storage at 80 °C. We measured viral titer as the median tissue culture infective dose (TCID_50_) per mL. The H1299 cells were seeded using 5 × 10^3^ cells per well in 96-well plates in 100 μL of maintenance media. After an overnight incubation, the cultured media was replaced with 100 μL of fresh media. Cells were incubated with 50 μL of diluted virus stock ranging from 10^–1^ to 10^–7^-fold. After incubation at 37 °C for another 7 days, the CPE was inspected under an inverted microscope to calculate TCID_50_/mL.

### 2.5. HCoV Infection

Cells were seeded at a density of 1 × 10^6^ cells per well in 6-well plates and incubated overnight at 37 °C. After three washes with serum-free media, cells were inoculated with 1 mL serum-free media containing HCoV at the indicated multiplicity of infection (MOI) for 1 h at 37 °C. The 0 h post infection (hpi) samples were harvested immediately, while subsequent cell samples were washed three times with serum-free media before 2 mL of media with 2% FBS was added. Cells or cultured media were harvested at the indicated time points post infection. Cell viability of the harvested cells was determined using Countess II Automated Cell counter (Thermo Fisher Scientific).

### 2.6. Detection of Viral RNA by Real-Time PCR

Total cell RNA was extracted using the MagMAX *mir*Vana Total RNA Isolation Kit (Thermo Fisher Scientific) with KingFisher systems (Thermo Fisher Scientific). RNA was quantified using NanoDrop Lite Spectrophotometer (Thermo Fisher Scientific). Total RNA (1 μg) was reverse transcribed using the High-Capacity cDNA Reverse Transcription Kit (Thermo Fisher Scientific). The cDNA samples were used for quantitative TagMan PCR using TaqManTM Universal Master Mix II, no UNG (Thermo Fisher Scientific), in an QuantStudio™ 5 System (Thermo Fisher Scientific). The FAM-MGB-labeled TaqMan probes for the indicated genes were purchased from Thermo Fisher Scientific (Supplementary Table S1). The viral RNA level was normalized to GAPDH as a reference gene. The resulting data were analyzed using Excel in Office 365 and GraphPad Prism 8.

### 2.7. Western Blotting

Cell lysates were prepared in 50 mM HEPES pH 7.5, 200 mM NaCl, 1 mM DTT, 1% Triton-100, and protease inhibitors (Thermo Fisher Scientific). Protein concentration was determined using the Bradford protein assay (Bio-Rad, Hercules, CA, USA). Comparable amounts (~10 μg) of protein samples were loaded onto a 4–20% SDS-PAGE gel (Bio-Rad) and transferred to a nitrocellulose membrane (Bio-Rad). Proteins were detected using an anti-VCP antibody (1:3000) (Thermo Fisher Scientific, MA3-004) for detecting p97, an anti-GAPDH antibody (1:5000) (Cell Signaling Technology, Danvers, MA, USA 2188S) for detecting GAPDH, an anti-coronavirus antibody (1:500) (Santa Cruz Biotechnology, Dallas, TX, USA sc-65653) for detecting HCoV-229E nucleocapsid protein, and an anti-coronavirus antibody (1:500) (MilliporeSigma, MAB9012) for detecting HCoV-OC43 nucleocapsid protein. Primary antibodies were detected using HRP-labeled goat anti-mouse or anti-rabbit antibodies (Bio-Rad). Blots were developed using Immobilon Western Chemiluminescent HRP Substrate (MilliporeSigma) and visualized using ChemiDoc MP Imaging System (Bio-Rad). Quantification was performed using Image Lab 6.0.1 (Bio-Rad).

### 2.8. p97 Inhibitor Treatment

Cells were treated with the indicated concentration of CB-5083 and infected with the virus. After 1 h of infection, cells were washed three times with serum-free media and then returned to media containing 2% FBS and the same concentration of CB-5083. Cultured media was replaced with fresh media at 8 hpi and harvested at 24 hpi for viral titer titration.

### 2.9. Cellular Viability

Cells were seeded using 2 × 10^5^ cells per well in 96-well plates and incubated overnight at 37 °C. Cells were treated with the indicated concentration of CB-5083 and infected with virus or mock infected. After 1 h of infection, cells were washed three times with serum-free media and then cultured with media containing 10% FBS and the same concentration of CB-5083. Cultured media was replaced with fresh media at 8 hpi. At 72 hpi, cellular viability was measured by a CellTiter-Glo^®^ Luminescent Cell Viability Assay (Promega, Madison, WI, USA).

### 2.10. Stage-Limited Inhibition Assay

H1299 cells were treated with 0.1% DMSO, 1 μM CB-5083, or 2 μM of NMS-873 from 0.5 h prior to virus infection until 8 h post-infection. After infection, cells were washed three times with serum-free media and then returned to media containing 2% FBS, 0.1% DMSO, 1 μM CB-5083, or 2 μM of NMS-873. Cells were collected at 8 hpi for viral RNA determination.

### 2.11. TMT Label Proteomics

Cell lysates were prepared in an 8 M urea lysis buffer which was prepared by dissolving urea in the buffer containing 20 mM HEPES pH 7.5, 100 mM NaCl, protease inhibitor (Thermo Fisher Scientific) and 100 μM MG132 (Selleckchem, Houston, TX, USA). The protein concentration was measured by Bradford. After reduction with 10 mM TCEP (Thermo Fisher Scientific) and alkylation with 25 mM 2-chloroacetamide (MP Biomedicals), the proteins were precipitated by adding six volumes of pre-chilled acetone and incubated at −20 °C overnight. Proteins were collected by high-speed centrifugation. The dry protein pellets were resuspended in 100 mM TEAB buffer containing Lys-C (Wako Chemicals, Irvine, CA, USA) and Trypsin (Thermo Fisher Scientific) to digest at 37 °C overnight. The peptide concentration was determined by a Quantitative Fluorometric Peptide Assay (Thermo Fisher Scientific). The 10 μg peptide of each sample was transferred and labeled with TMTpro 16plex reagents (Thermo Fisher Scientific) by following the manufacture’s instruction. Then, 10 μg peptide of a same amount mixture of all samples was labeled with the same channel at each TMT set to be a bridge of different TMT sets. Labeled samples were combined and dried using vacuum centrifugation. Samples were then separated into eight fractions using the High pH reversed-phase peptide Fractionation Kit (Thermo Fisher Scientific). The fractions were dissolved with 0.1% FA and peptide concentration was determined with Quantitative Colorimetric Peptide Assay (Thermo Fisher Scientific).

The TMT labeling LC-MS/MS experiments were performed using an EASY-nLC 1000 connected to an Orbitrap Eclipse Tribrid mass spectrometer. For this, 1 μg of sample was loaded onto an Aurora UHPLC Column and separated over 136 min at a flow rate of 0.4 μL/min with the following gradient: 2–6% Solvent B (7.5 min), 6–25% B (82.5 min), 25–40% B (30 min), 40–95% B (1 min), and 95% B (15 min). Solvent A consisted of 97.8% H_2_O, 2% ACN, and 0.2% formic acid, and solvent B consisted of 19.8% H_2_O, 80% ACN, and 0.2% formic acid. An MS1 scan was acquired in the Orbitrap at 120 k resolution with a scan range of 350–1800 m/z. The AGC target was 1 × 10^6^, and the maximum injection time was 50 ms. Dynamic exclusion was set to exclude features after 1 time for 45 s with a 5-ppm mass tolerance. The MS2 scans were acquired with the HCD activation type with the Orbitrap. The isolation window was 0.5 m/z, the collision energy was 32%, the maximum injection time was 86 ms, and the AGC target was 5 × 10^4^. Ion source settings were as follows: ion source type, NSI; spray voltage, 1650 V; ion transfer tube temperature, 300 °C. System control and data collection were performed by Xcalibur v.4.0.

### 2.12. Proteomic Data Processing

The proteomic analysis was performed with the Proteome Discoverer 2.4 (Thermo Fisher Scientific) using the SequestHT search algorithm with Percolator validation and the UniProt human (UP000005640), HCoV-229E (UP000006716), and HCoV-OC43 (UP000007552) databases. Normalization was performed relative to the total peptide amount. Volcano plots and heatmaps were generated with Prism 8. A set of proteins that were significantly up- or down-regulated between cells with HCoV or mock infection at each time point were identified as differentially expressed proteins (DEPs) (|log2 FC| > 0.3, Student’s *t* test, *p* < 0.05). Protein abundance in infected cells with control shRNA or p97 shRNA was further normalized to that in corresponding mock-infected cells. A set of proteins that were significantly up- or down-regulated between cells with control shRNA and p97 shRNA at each time point after infection were identified as DEPs (|log2 FC| > 0.3, Student’s *t* test, *p* < 0.05). The DEPs from each point were selected for multi-set Reactome pathway enrichment analysis using a web-based portal Metascape [22].

A set of proteins with significant up or down effects (Student’s *t* test, *p* < 0.05) in the infected samples compared with the mock control were selected and clustered by using the default setting in Cluster 3.0. and Java TreeView 1.2.0. Protein clusters were increased over time after virus infection and were selected for overlap analysis. Venn plots were generated with FunRich 3.1.4 [23,24,25]. A set of proteins from each infection were tested for enrichment of Reactome pathways [26] using a web server g:Profiler [27]. Significant terms were identified that have a *p*-adjusted value < 0.05 after Benjamin–Hochberg correction. Bubble plots were generated using the R package ggplot2 3.3.3 [28]. Proteins in the same enriched pathways were mapped to a web-based search tool STRING [29], to acquire protein–protein interaction networks. Proteomic analysis results can be found in Supplementary Tables S2–S5.

## 3. Results

### 3.1. H1299 Is Susceptible to Both HCoV-229E and HCoV-OC43 Infection

**Figure 1:**
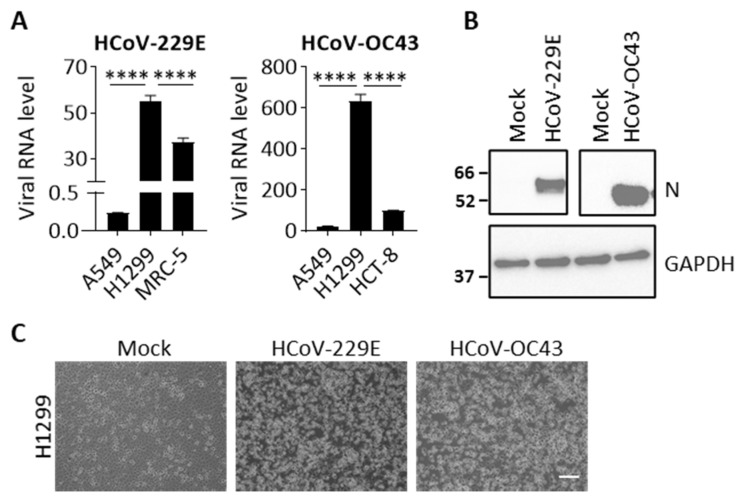
HCoV-229E and HCoV-OC43 infection in human cells. (**A**) Cell lines, including A549, H1299, MRC-5, and HCT-8 were infected with HCoV-229E (MOI 0.05) or HCoV-OC43 (MOI 0.01). Viral RNA in cell lysates was quantified by real-time PCR at 24 hpi. Data show viral RNA levels relative to GAPDH. Error bars represent SD (*n* = 3). Statistical analysis was conducted with one-way ANOVA: ****, *p* < 0.0001. (**B**) Representative western blot images showing the expression viral N protein and GAPDH (loading control) in cell lysates at 24 hpi. Full size images are presented in Supplementary Figure S1. (**C**) Representative images show CPEs in the HCoV-229E or HCoV-OC43-infected H1299 cells at 48 hpi. Scale bar, 10 μm.

To firstly identify cell lines that can be infected by both HCoV-229E and HCoV-OC43 viruses we evaluated four cell lines including two lung cancer cell lines, A549 and H1299, as well as the two virus propagating cell lines, MRC-5 and HCT-8. At 24 hpi, cells were harvested to determine viral RNA levels. Viral RNA was not detectable in cells with mock infection. As shown in Figure 1A, a relatively higher viral RNA level was detected in H1299 than in A549 cells, which are 230- and 30-fold for HCoV-229E and HCoV-OC43, respectively. Noticeably, the viral RNA levels in H1299 cells were even higher than the corresponding propagated cells. We further confirmed whether viral proteins are expressed in H1299 cells after infection. Figure 1B shows that viral nucleoprotein (N) protein was detected in HCoV-229E or HCoV-OC43 infected cells at 24 hpi. Moreover, we also observed severe CPEs such as cell floating and lysis in H1299 cells that were infected with either HCoV-229E or HCoV-OC43 at 48 hpi (Figure 1C), indicating that the viral infection progresses rapidly and robustly in H1299 cells. Therefore, we selected the permissive H1299 cell line as a model for both HCoV-229E and HCoV-OC43 infection in subsequent experiments.

### 3.2. p97 Is a Potential Therapeutic Target for HCoV Infection

**Figure 2:**
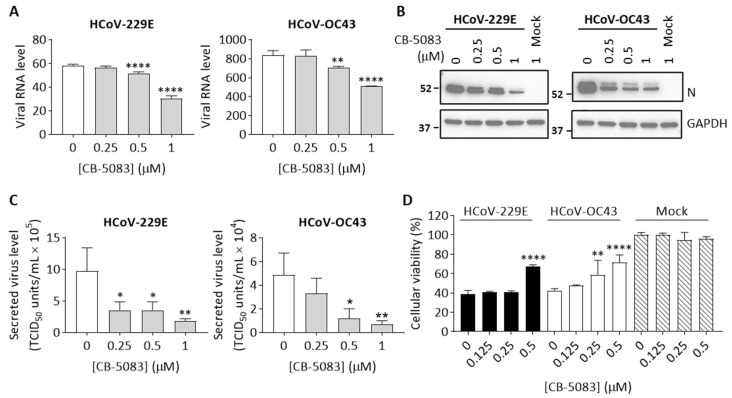
p97 inhibition by CB-5083 suppresses HCoV replication in H1299 cells. H1299 cells were infected with HCoV-229 (MOI 0.5), HCoV-OC43 (0.01), or mock-treated in the presence of CB-5083 at the indicated concentration. Cultured media containing CB-5083 was removed at 8 hpi and replaced with fresh media without CB-5083. (**A**) Cells were harvested to quantify viral RNA levels in cell lysates at 24 hpi. Data shows viral RNA levels relative to GAPDH. (**B**) Representative Western blot analysis of the expression viral N protein and GAPDH (loading control) in cells treated with CB-5083 at 24 hpi after HCoV or mock infection. Full-length blots are included in Supplementary Figure S3 (**C**) Titer of secreted virus in culture media collected from the HCoV-infected cells at 24 hpi was quantified by determining TCID_50_ in H1299 cells. (**D**) Cellular viability of infected cells was determined at 72 hpi and normalized to mock-infected and DMSO-treated controls. All conditions were performed in biological triplicate. Error bars represent SD (*n* = 3). Statistical analysis was conducted with one-way ANOVA compared to infected control (0 μM of CB-5083): *, *p* < 0.05; **, *p* < 0.01; ****, *p* < 0.0001.

To evaluate whether p97 is an effective therapeutic target for treating HCoV infection, we tested the effects of p97 inhibition on HCoV replication in H1299 cells by using a p97 inhibitor CB-5083 to directly inhibit p97 activity [30]. The H1299 cells were infected with HCoV-229E or HCoV-OC43 in the presence of different doses of CB-5083 (0.25, 0.5, 1 μM) for 1 h. After infection, the cells were grown in media containing CB-5083 until 8 hpi. Cells were harvested at 24 hpi with good conditions and with similar cell viability (Supplementary Figure S2). Figure 2A shows that HCoV-229E and HCoV-OC43 viral RNA levels were reduced by CB-5083 in a concentration-dependent manner. Notably, CB-5083 at 1 μM significantly reduced levels of HCoV-229E and HCoV-OC43 viral RNA by ~47% and ~40%. Of note, the expression level of viral N protein in cells was in response to different concentration of CB-5083, showing dose-dependent effects of CB-5083 treatment (Figure 2B). In addition, we also harvested the culture media at 24 hpi to quantify the titer of the progeny virus. As shown in Figure 2C, CB-5083 treatment effectively reduced progeny virus levels. The viral titers of secreted HCoV-229E and HCoV-OC43 were significantly decreased by ~81% and ~85%, respectively, in the presence of 1 μM CB-5083. These results confirm that p97 inhibition suppresses HCoV replication in H1299 cells.

We further determined whether CB-5083 is capable of preventing cell death caused by the HCoV infection. The H1299 cells were infected with HCoV-229E, HCoV-OC43, or mock-treated in the presence of several concentrations of CB-5083. Cellular viability was examined at 72 hpi. We normalized results for each infection to the mock-infected and DMSO-treated control cells. As shown in Figure 2D, the tested concentrations of CB-5083 up to 0.5 μM did not change H1299 cellular viability in mock-treated cells. Notably, CB-5083 (0.5 μM) significantly increased cellular viability by ~29% compared to the HCoV-infected and DMSO-treated control for both HCoV229E and HCoV-OC43 infection, indicating that p97 inhibition protects cells from HCoV infection. Taken together, the data show the potential of p97 as the therapeutic target for treating HCoV infection, indicating the antiviral activity of the p97 inhibitor CB-5083.

### 3.3. p97 Is Required for Early Stages of HCoV Replication

**Figure 3:**
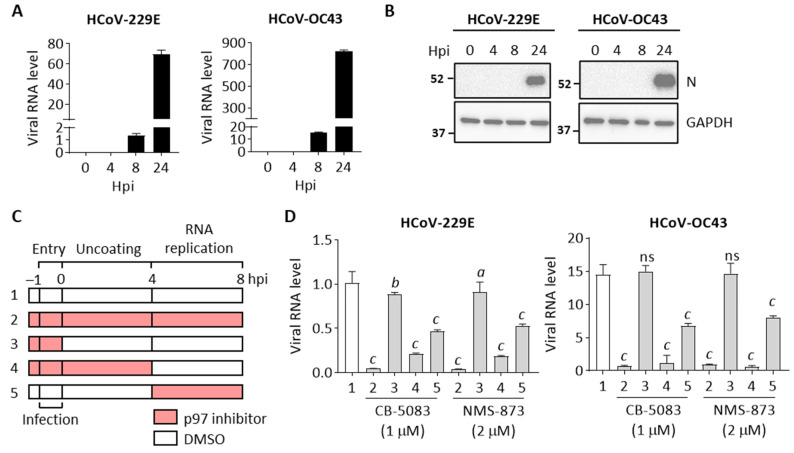
p97 is essential for early stages of HCoV replication. H1299 cells were infected with HCoV-229E (MOI 0.5) or HCoV-OC43 (MOI 0.01). (**A**) Quantification of viral RNA in cell lysates over time after infection. Data shows RNA levels relative to GAPDH. (**B**) Representative Western blot showing viral N protein and GAPDH expression in the infected cells. Full bot images are depicted in Supplementary Figure S5. (**C**) H1299 cells were continuously treated with DMSO or p97 inhibitors (CB-5083 and NMS-873) at the indicated period from 30 min before infection to 8 hpi and harvested to determine viral RNA levels. All conditions were performed in biological triplicate. (**D**) Quantification of viral RNA in the cell lysates harvested at 8 hpi. Data show relative RNA levels to GAPDH. Error bars represent SD (*n* = 3). Statistical analysis was conducted with one-way ANOVA compared to group 1: *a*, *p* < 0.01; *b*, *p* < 0.001; *c*, *p* < 0.0001; ns, no significance.

To determine the effects of p97 on different stages of the HCoV replication cycle, we used two p97 inhibitors, CB-5083 and NMS-873, to perform a stage-limited inhibition assay. We determined the time frame for the virus life cycle by measuring viral RNA levels in cells at four time points (0, 4, 8, and 24 hpi) after HCoV-229E or HCoV-OC43 infection. As shown in Figure 3A, both HCoV-229E and HCoV-OC43 RNA levels were similar at 0 hpi and 4 hpi and increased at 8 hpi by ~250-folds and ~280-folds, respectively. The viral RNA level sequentially increased at 24 hpi by ~12,000-fold and ~15,000-fold for HCoV-229E and HCoV-OC43, implying that virus entry occurs at -1 to 0 hpi, followed by uncoating at 0 to 4 hpi and RNA replication occurs at 4 to 8 hpi. We also confirmed the expression of viral N protein at the four time points. Figure 3B shows that viral N protein was only detected in the HCoV-infected cells at 24 hpi, indicating that transcription of viral structure proteins starts after 8 hpi.

Thus, to determine the impact of p97 at the early stage of virus replication, cells were vehicle treated (DMSO) or treated with two p97 inhibitors, CB-5083 or NMS-873, at different timepoints post infection, and then harvested at 8 hpi to analyze viral RNA levels (Figure 3C). No significant changes were seen in cell viability of cells treated with DMSO (group 1) or inhibitors throughout the experiment (group 2) in the HCoV-infected cells (Supplementary Figure S4). Extensive reduction in viral RNA levels was observed in cells incubated with inhibitors treated from pretreatment to 8 hpi (group 2) in both HCoV-229E and HCoV-OC43 infected cells compared to the vehicle treated control (group 1) with similar cell viability (Figure 3D). Importantly, early treatment (group 3: pretreatment to 0 hpi) caused only minor or no effects on viral RNA levels. However, significant reductions in viral RNA levels were observed in cells treated with inhibitors from pretreatment to 4 hpi (group 4), in which viral RNA levels were reduced by 79–82% and 92–96% for HCoV-229E and HCoV-OC43 infection, respectively, suggesting that p97 is essential for HCoV uncoating but may not be important for early stages of viral infection such as receptor binding and entry (Figure 3D). Moreover, p97 inhibition at 4 to 8 hpi (group 5) also resulted in decreased viral RNA levels by 54–48% and 53–45% for HCoV-229E and HCoV-OC43 infection, respectively (Figure 3D), indicating that p97 is also involved in processes linked to viral RNA replication. Altogether, these results confirm that p97 plays important functions at the early stages of the virus life cycle, including in virus uncoating and viral RNA replication.

### 3.4. Effects of p97 on Multiple Pathways after HCoV Infection

To complement the results from p97 inhibitors with genetic inhibition and to understand additional roles of p97 during the whole virus life cycle, we determined the impact of p97 depletion on host responses after HCoV infection. The p97 protein expression was knocked down using shRNA because p97 is an essential gene. This p97 depletion may have pleiotropic effects in cells and be lethal given its essential roles in multiple cellular processes [13]. We therefore used an inducible knockdown system to abolish p97 function as described previously [21]. We established stably transfected cells with Tet-regulated expression of p97-specific shRNA, or a control shRNA, using lentiviral infection. Immunoblot analysis confirmed that p97 expression was reduced by 90% in the presence of 0.5 μg/mL Dox for 72 h and we extended the time to 96 and 120 h to ensure cells are in good condition (Supplementary Figure S6).

**Figure 4:**
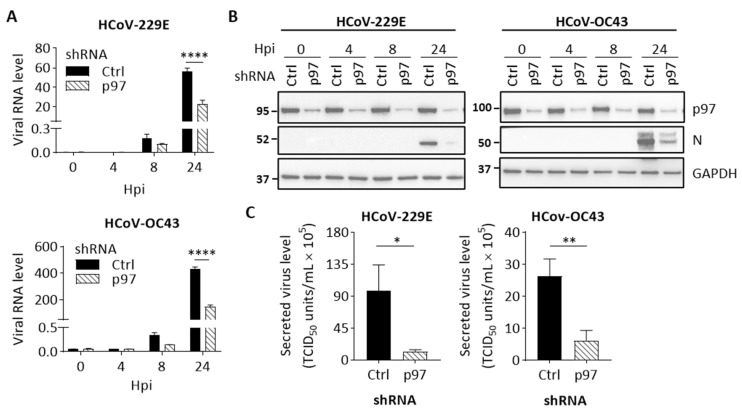
p97 knockdown suppresses HCoV replication in H1299 cells. H1299 cells with inducible control shRNA (Ctrl shRNA) or p97 shRNA were inoculated with Dox (0.5 μg/mL) for 72 h. Cells were infected with HCoV-229E (MOI 0.05) or HCoV-OC43 (MOI 0.01). After 1 h of infection, cells were washed and harvested at 0, 4, 8, or 24 hpi. Mock-infected cells were harvested at 24 hpi. (**A**) Quantification of viral RNA in cell lysates over time after infection. Data shows viral RNA levels relative to GAPDH. (**B**) Representative western blot images showing the expression of p97, viral N protein, and GAPDH (loading control) in cell lysates across the infection time course. Full-length blots can be found in Supplementary Figure S8. (**C**) Viral titer in culture media collected from HCoV-infected cells at 24 hpi was quantified by determining TCID_50_ in H1299 cells. Error bars represent SD (*n* = 3). Statistical analysis was conducted with two-way ANOVA for Figure 4A and Student’s *t* test for Figure 4C: *, *p* < 0.05; **, *p* < 0.01; ****, *p* < 0.0001.

We then characterized the effects of p97 depletion on HCoV replication. Cells with control shRNA or p97 shRNA were induced using Dox for 72 h and followed by HCoV-229E, HCoV-OC43, or mock infection. Cells infected by HCoV were harvested at 0, 4, 8, or 24 hpi or at 24 hpi after mock infection. Cell viability analysis shows no significant difference between cells harvested at 24 hpi after HCoV or mock infection (Supplementary Figure S7). We subsequently measured viral RNA levels in HCoV-infected cells. As shown in Figure 4A, p97-knockdown cells displayed significantly lower viral RNA levels normalized to GAPDH than controls. The HCoV-229E and HCoV-OC43 RNAs were present at 58% and 66%, respectively, relative to controls at 24 hpi, indicating that p97 is important for HCoV replication. We also confirmed the cellular levels of viral N protein over time after infection by immunoblot analysis. Figure 4B shows that a reduction in viral N protein levels was observed in p97 knockdown cells for HCoV-229E or HCoV-OC43-infected cells at 24 hpi. Importantly, p97 depletion was efficient, as confirmed by the low expression levels observed across the infection time course (Figure 4B). Next, to examine the effects of p97 on the ability to produce progeny virions from HCoV-infected cells, a viral titer in the culture media was quantified at 24 hpi by determining TCID_50_. As shown in Figure 4C, p97 depletion resulted in a significant reduction in secreted viral titer by 87% for HCoV-229 and 76% for HCoV-OC43. Collectively, we demonstrate that impairment of p97 function by inhibiting its expression effectively suppresses both HCoV-229E and HCoV-OC43 replication in H1299 cells, indicating that p97 is required for HCoV replication.

**Figure 5:**
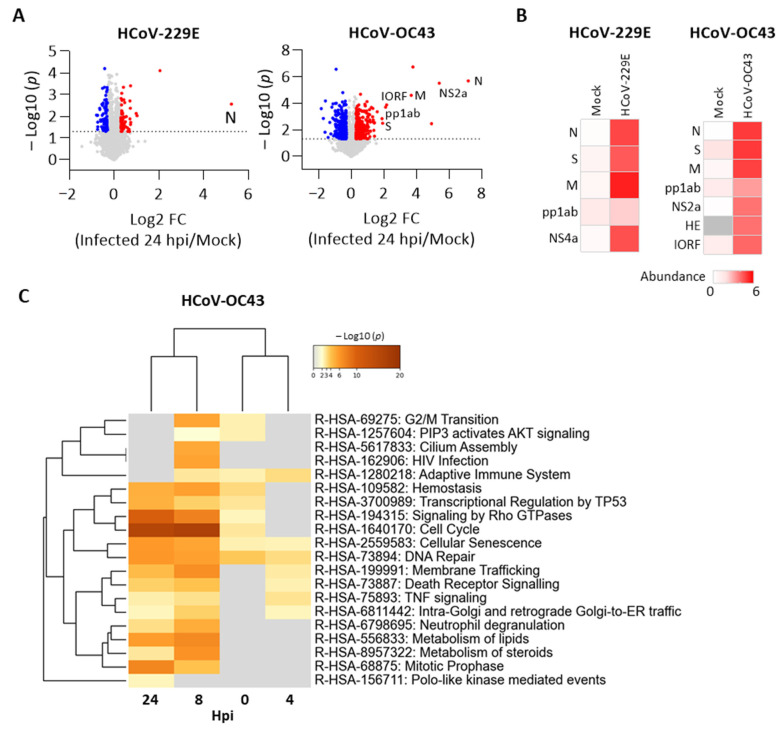
Cellular pathway changes in H1299 cells after HCoV-OC43 infection. Proteomic analysis of H1299 cells in response to HCoV-229E or HCoV-OC43 infection. (**A**) Volcano plots of the change in total protein levels comparing infected cells 24 hpi vs. mock infection. Up- (red) and down-regulated proteins (blue) with |log2 FC| > 0.3 and −log10 *p* value > 1.3 are highlighted. Detected viral proteins are labeled. (**B**) Protein abundance of all detected viral proteins across the infection time course. (**C**) Multi-list Reactome enrichment of DEPs (|log2 FC| > 0.3, *p* < 0.05) at each time point after HCoV-OC43 infection. Data present the top 20 statistically enriched Reactome pathway terms (–log10 *p* value > 1.3).

We further performed a proteomic analysis of cells with or without p97 knockdown after HCoV-229, HCoV-OC43, or mock infection. Proteomes from two sets of cells infected with HCoV-229E or HCoV-OC43 were analyzed separately. We identified 8236 proteins in the HCoV-229E set, of which five are HCoV-229E viral proteins. We identified 8830 proteins in the HCoV-OC43 set, of which seven are HCoV-OC43 viral proteins. To understand the host responses during HCoV replication, we compared the fold change of protein level to identify DEPs (|log2 FC| > 0.3, *p* < 0.05) between cells infected by HCoV and mock at 24 hpi. As shown in Figure 5A, DEPs were identified in HCov 229E infection, of which HCoV-229E N protein was significantly increased. Of the increased DEPs, HCoV-OC43 viral proteins were significantly upregulated, including N, spike glycoprotein (S), membrane protein (M), non-structural protein 2a (NS2a), replicase polyprotein 1ab (pp1ab), and protein I (IORF). Increased viral protein abundance in the infected cells compared to mock infection also confirmed viral infection (Figure 5B). We further identified DEPs (|log2 FC| > 0.3, *p* < 0.05) from each time point over the infection time course for HCoV-229E or HCoV-OC43 and applied for multi-list pathway enrichment analysis. No statistically significant results were observed for the HCoV-229E infection due to the fact that relatively few DEPs were detected. In contrast, DEPs from the HCoV-OC43 sample set were enriched in several pathways. Of note, cellular senescence and DNA repair were enriched across the whole period for HCoV-OC43 (Figure 5C). These data show the host responses caused by the HCoV-OC43 infection, which may be favored for HCoV replication.

We also investigated the effects of HCoV infection on cells and tested whether HCoV-229E, HCoV-OC43, and SARS-CoV-2 induce similar changes in cellular processes in infected cells. We compared shifts in pathways in response to the virus infection in H1299 cells treated with control shRNA. In addition to HCoV-229 and HCoV-OC43, proteome results from SARS-CoV-2 infected Caco-2 cells were included [31]. We employed hierarchical clustering to identify proteins that increased over time after each virus infection (Supplementary Figure S9A). Individual pathway enrichment analysis reveals that proteins in each cluster were enriched in the same pathways, including cellular responses to stress, host interactions of HIV factors, and mitotic anaphase (Supplementary Figure S9B). That these pathways overlap indicates which host machineries are required for HCoV replication. We further analyzed protein–protein interaction between the three groups of proteins enriched in the same pathways. Proteins from three infections in the same pathways showed overlapping and direct interaction with each other (Supplementary Figures S10–S12), particularly PSMB7 (proteasome subunit beta type-7) was identified in three pathways from the overlap of HCoV-OC43 and SARS-CoV2 infection proteomes, indicating that the overlapping proteins may be modulated during infection by different strains of HCoV.

**Figure 6:**
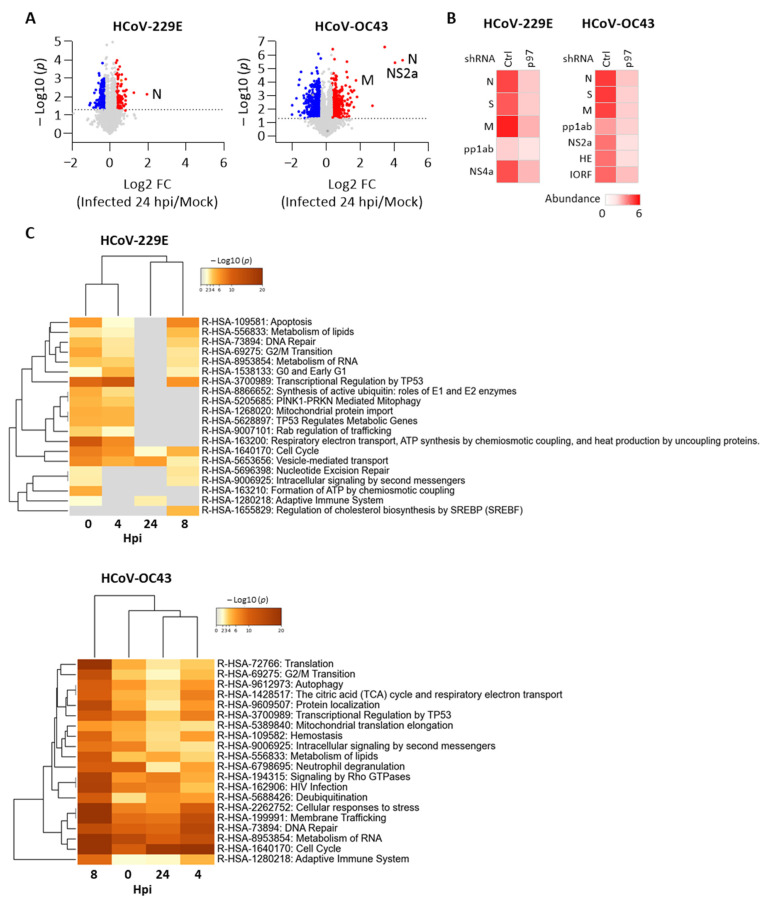
p97 depletion impacts host cell cycle regulators during HCoV infection. Proteomic analysis of H1299 cells with control shRNA (Ctrl shRNA) or p97 shRNA after HCoV-229E or HCoV-OC43 infection. (**A**) Volcano plots of the change in total protein levels comparing HCoV infected cells with control shRNA vs. p97 shRNA at 24 hpi. Up- (red) and down-regulated proteins (blue) with |log2 FC| > 0.3 and −log10 *p* value > 1.3 are highlighted. Detected viral proteins are labeled. (**B**) Abundance of detected viral proteins in HCoV-infected cells with control or p97 shRNA at 24 hpi. (**C**) Multi-list Reactome enrichment of DEPs (|log2 FC| > 0.3, *p* < 0.05) between infected cells with control or p97 shRNA at each time point after HCoV-229E or HCoV-OC43 infection. Data presents the top 20 statistically enriched Reactome pathway terms (–log10 *p* value > 1.3).

Next, we examined global changes in the host proteome over time between cells treated with p97 vs. control shRNA after HCoV infection. Protein abundance in infected cells with p97 or control shRNA was normalized to that in corresponding mock-infected cells to verify virus effects on each cell line. We then compared the fold change of the normalized protein levels between cells with control shRNA and p97 shRNA at 24 hpi to identify DEPs (|log2 FC| > 0.3, *p* < 0.05). Figure 6A shows the detected DEPs in both HCoV 229E and HCoV-OC43 infection, indicating the HCoV infection resulted in significant changes in the cells with or without p97 expression. We also compared viral protein abundance in cells. As expected, lower levels of viral proteins were observed in the p97-knockdown cells for both HCoV-229 and HCoV-OC43 infection (Figure 6B). Consistent with the above results, this data shows that p97 depletion reduced the level of viral proteins in cells after infection, suggesting a pivotal role for p97 in both HCoV-229E and HCoV-OC43 replication. To uncover the functional effect of host responses during infection, we performed a multi-list pathway enrichment analysis of the DEPs between infected cells with control and p97 shRNA across the infection time course. Figure 6C shows the top 20 statistically enriched pathway terms across the whole period for HCoV-229E or HCoV-OC43 infection. Of note, the DEPs were enriched in the cell cycle and vesicle-medicated transport during HCoV-229E replication. In contrast, relatively more terms were enriched for the HCoV-OC43 infection, including cell cycle-replated pathways. This suggests that p97 depletion affects multiple pathways during HCoV replication, in which cell cycle is the common pathway identified in both HCoV-229E and HCoV-OC43 infection. Collectively, this data shows the extensive p97-dependent host processes, particularly cell cycle related pathways that may support HCoV infection.

## 4. Discussion

The p97 function is involved in regulating HCoV replication. The p97 depletion by siRNA leads to decreased double-stranded RNA, a hallmark of viral transcription/replication, and lower viral genomic RNA and N protein levels, which is reflected in reduced secreted virus particles in the HCoV-229E infected Huh7 cells [32]. Importantly, p97 knockdown prevents infectious bronchitis virus, the coronavirus of chicken, from exiting the endosome into the cytoplasm during early steps of virus replication [32]. In addition, p97 is also required for massive viral protein production in infected cells at later stage of SARS-CoV-2 replication [28]. Analysis of proteome changes after SARS-CoV-2 infection in Caco-2 cells shows that proteins of the proteostasis machinery acted in a comparable manner to the viral proteins [28]. It is important to note that p97 is a key factor of protein homeostasis, which is important for high translation rates of viral proteins in the infected cells. Inhibition of p97 function by NMS-873, an allosteric and non-ATP-competitive inhibitor of p97, effectively suppresses SARS-CoV-2 replication in Caco-2 cells [31]. Moreover, p97 is also directly involved in host responses regulated by SARS-COV-2 proteins. While the expression of SARS-CoV-2 membrane-associated protein ORF9c in A549 cells resulted in downregulation of proteins involved in host immune responses, such as interferon signaling and antigen processing, p97 inhibition by NMS-873 effectively blunted these effects in ORF9c-expressing cells [33], suggesting that that ORF9c ability to attenuate antiviral responses requires p97. Therefore, p97 is essential for viral replication as well as viral immune evasion in host cells.

The HCoVs are highly dependent on host cell pathways for replication. We found proteome changes in cellular senescence related proteins during the HCoV-OC43 replication at a 24-h period. In addition, we observed significant changes of the proteins in the cell cycle at the late stage of HCoV-OC43 replication. Importantly, our data shows common proteome changes in mitotic anaphase regulation after infection with HCoV-229E, HCoV-OC43, and SARS-CoV-2. Therefore, host cell cycle changes may affect viral replication. Similarly, HCoV-229E has been showed to cause transcriptomic changes in several pathways that are linked to the cell cycle, including control of chromosomal replication, cyclins and cell cycle regulation, and cell cycle G1/S checkpoint [34]. Not limited to HCoV-229E, it was found that cell cycle arrest occurs at S/G2 in cells infected with SARS-CoV-2 [17]. Moreover, the study also shows the role of p97 in regulating cell cycle progression and chromatin-associated functions by interacting with regulators in the cell cycle [35]. Of note, our data show proteome changes in cell cycle related proteins in the presence or absence of p97 after infection with HCoV-229E and HCoV-OC43. As such, p97 may be involved in cell cycle pathways triggered by HCoV infection, which seems to be a vital strategy that facilitates HCoV replication.

Interestingly, p97 also has been shown to regulate different stages of the viral life cycle for other enveloped RNA viruses. For example, p97 facilitates Sindbis virus entry in insects and mammals by regulating trafficking of the entry receptor NRAMP2 [36]. Moreover, p97 is required for nucleocapsid disassembly to release uncoated viral genome in the cytoplasm during the entry of the yellow fever virus [37]. In addition to the role during viral entry, p97 is also important for RNA replication. Perturbation of p97 expression decreases levels of the new synthesized West Nile virus genomic RNA [38]. Interestingly, p97 was observed to co-localize with non-structural replicase proteins [39]. Thus, p97 may regulate viral RNA replication by direct interaction with the viral replication complex. Likewise, we found that p97 is required for HCoV replication. Depletion of p97 suppresses HCoV-229E and HCoV-OC43 replication in H1299 cells. Importantly, our data indicate that p97 mainly affects virus uncoating and RNA replication, two events in the early stages of the HCoV life cycle. Although we were not able to determine whether p97 also regulates late stages of virus replication, p97 appears to contribute to replication of various RNA viruses through direct and indirect regulation, making it a promising target for the treatment of virus infection.

Given the essential role of p97 during virus replication, small molecule inhibitors of p97 are potential antiviral drugs. Both CB-5083 and NMS-873 are the most advanced p97 inhibitors to date. The ATP-competitive inhibitor CB-5083 has been used for solid tumor or multiple myeloma treatment in phase I trials [40]. Importantly, CB-5083 treatment inhibited Japanese encephalitis virus (JEV) RNA synthesis and viral titer secreted by infected cells [41]. The CB-5083 administration delays Japanese encephalitis symptoms caused by virus infection and also reduces virus titer in the brain in JEV-infected mice [41]. Similar effects were observed with the allosteric inhibitor NMS-873. The NMS-873 inhibits both influenza A and B viruses with EC_50_ at a non-cytotoxic concentration of 28–556 nM in human lung cell lines and primary cells [42]. In addition to these two known p97 inhibitors, one irreversible p97 inhibitor, LC-1310, also shows inhibitory ability to the replication of human cytomegalovirus and improves cell viability after infection [43]. In our study, we demonstrated that both NMS-873 and CB-5083 effectively inhibit HCoV-229E and HCoV-OC43 replication in the early steps of infection. Furthermore, CB-5083 effectively suppresses HCoV-229E and HCoV-229E RNA levels and virus secretion in infected cells. Importantly, CB-5083 also exhibits protective effects against HCoV-229E and HCoV-OC43 infection, reducing CPEs induced by the virus. Therefore, p97 inhibitors are a potential broad-spectrum antiviral drug candidate, that can potentially be used to target coronaviral family members.

## 5. Conclusions

In this study, we find that p97 plays an important role in the life cycle of HCoV. We show that p97 inhibition effectively suppresses virus replication and cell death caused by the infection of HCoV-229E or HCoV-OC43 in H1299 cells. Moreover, stage-limited inhibition assays using p97 inhibitors suggest that p97 particularly impact early stages in the viral life cycle, including virus uncoating and RNA replication. Interestingly, significant changes occur in host proteins linked to the cell cycle across different sates of the viral replication cycle between cells with or without p97 depletion after HCoV-229E or HCoV-OC43 infection. This suggests that coronaviruses may affect the host cell cycle in a p97-dependent manner. Overall, this data indicates that p97 depletion or inhibition impacts host machines that favor virus replication and also provides evidence that p97 is a promising therapeutic target for the HCoV infection.

It will therefore be important to move forward and further test the potential for p97 inhibitors to improve therapeutic outcomes of viral infection across virus families. Such broad inhibitors have the potential to be useful in therapeutics for newly emerging viruses.

## Data Availability

All relevant data generated during this study are included in the article and the supplementary information. The mass spectrometry raw data are deposited to the ProteomeXchance Consortium (https://www.ebi.ac.uk/pride/ (accessed on 21 October 2021)) via the PRIDE repository with the dataset identifier PXD026216 and 10.6019/PXD026216). Additional raw data generated during the current study and relevant information are available from the corresponding authors upon request.

## Supplementary Materials

The following are available online at https://www.mdpi.com/article/10.3390/cells10112953/s1, Figure S1: Full blot images of results shown in Figure 1B; Figure S2: Cell viability of HCoV-infected H1299 cells in the presence of CB-5083; Figure S3: Full blot images of results shown in Figure 2B; Figure S4: Cell viability of HCoV-infected H1299 cells in the stage-limited inhibition assay; Figure S5: Full blot images of results shown in Figure 3B; Figure S6: Knockdown of p97 expression in H1299 cells; Figure S7: Cell viability of H1299 cells with inducible control shRNA (Ctrl shRNA) or p97 shRNA after HCoV infection; Figure S8: Full blot images of results shown in Figure 4B; Figure S9: Overlapping host responses in cells after HCoV-229E, HCoV-OC43, or SARS-CoV-2 infection; Figure S10: Proteins enriched in “Cellular responses to stress”; Figure S11: Proteins enriched “Host interactions of HIV factors”; Figure S12: Proteins enriched “Mitotic anaphases”; Table S1: TagMan probes list; Table S2: Proteomics of H1299 cells with control shRNA and p97 shRNA after HCoV-229E infection; Table S3: Proteomics of H1299 cells with control shRNA and p97 shRNA after HCoV-OC43 infection; Table S4: Reactome pathway enrichment analysis; Table S5: Multi-list Reactome pathway enrichment analysis.
