# Supplementary material for: Impacts of p97 on Proteome Changes in Human Cells during Coronaviral Replication
Source: Cells. 2021 Oct 29;10(11):2953. doi: 10.3390/cells10112953 (PMC8616207; doi:10.3390/cells10112953)
Supplement: Supplementary file 1 [file cells-10-02953-s001.zip › Supplementary materials.pdf]

# Supplementary Materials

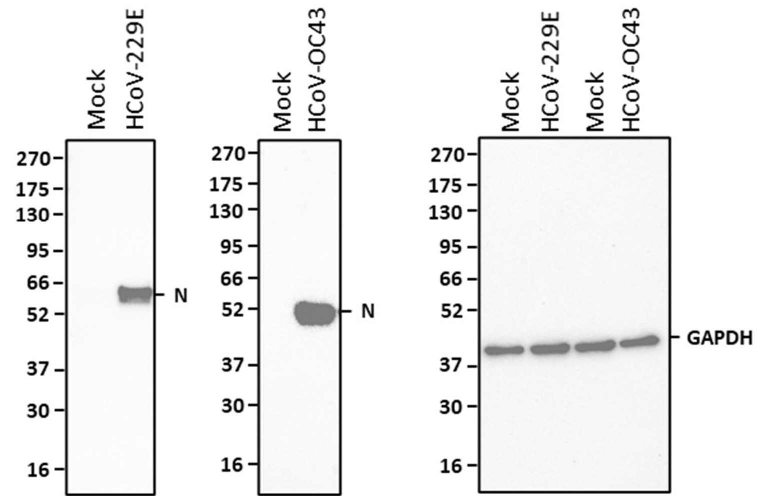


**Figure S1.** Full blot images of results shown in Figure 1B.

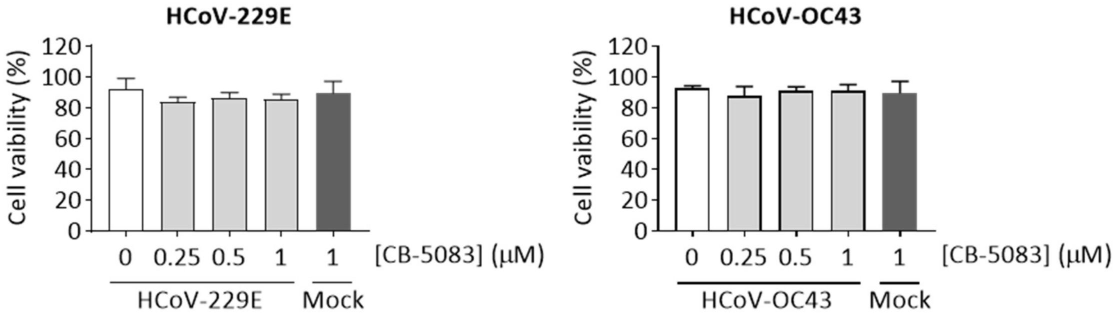


**Figure S2.** Cell viability of HCoV-infected H1299 cells in the presence of CB-5083. H1299 cells were infected with HCoV-229 (MOI 0.5), HCoV-OC43 (0.01), or mock in the presence of CB-5083 at the indicated concentration (0, 0.25, 0.5, 1 mM). Cultured media containing CB-5083 was removed at 8 hpi and replaced with fresh media without CB-5083. Cells were harvested at 24 hpi to quantify viral RNA level and viral N protein expression as shown in Figure 2A and Figure 2B. Cell viability of the harvested cells was determined using Countess II Automated Cell counter. Error bars represent SD ($n$ = 3). No statistical significance was identified between groups using one-way ANOVA.

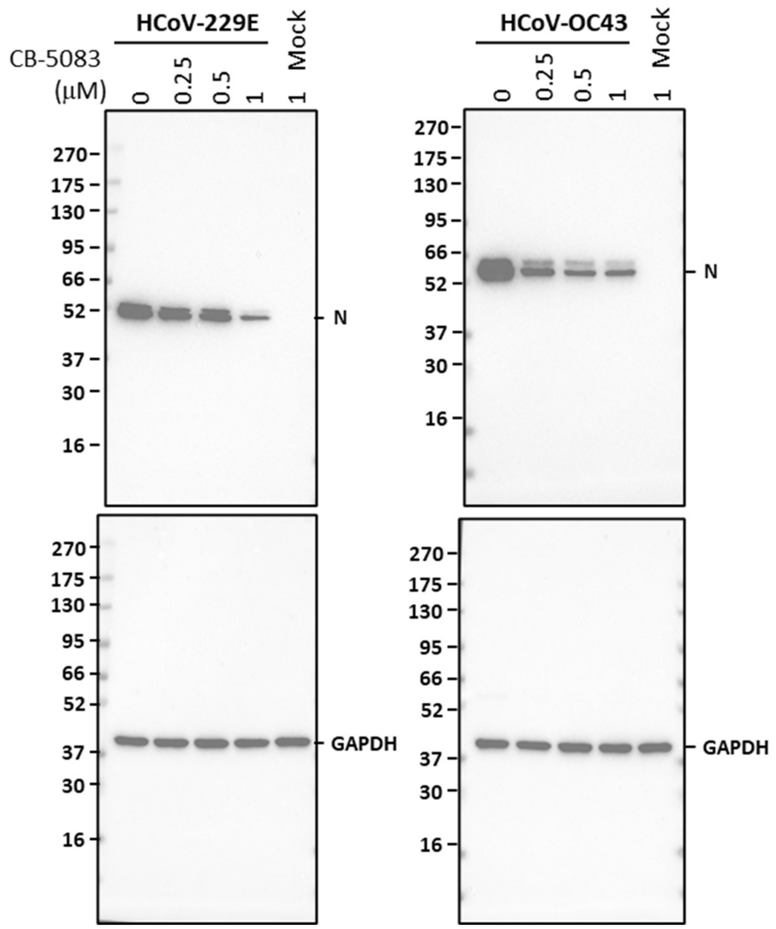


**Figure S3.** Full blot images of results shown in Figure 2B.

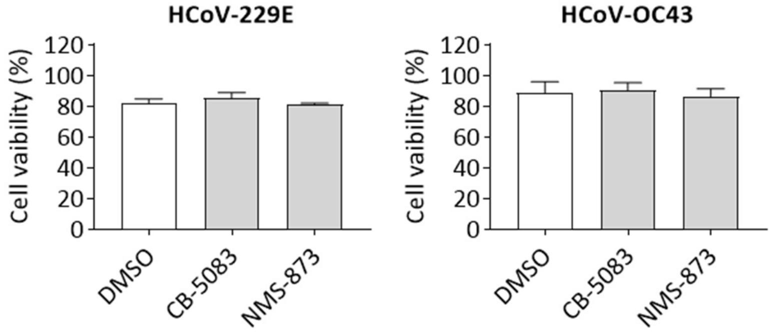


**Figure S4.** Cell viability of HCoV-infected H1299 cells in the stage-limited inhibition assay. H1299 Cells were continuously treated with DMSO or p97 inhibitors, CB-5083 (1 μM) and NMS-873 (2 μM), from -0.5 h prior to virus before infection to 8 hpi Cells were infected with HCoV-229E (MOI 0.5) or HCoV-OC43 (MOI 0.01) and harvested at 8 hpi to quantify viral RNA level as shown in Figure 3D. Cell viability of the harvested cells was determined using Countess II Automated Cell counter. Error bars represent SD ($n$ = 3). No statistical significance was identified between groups using one-way ANOVA.

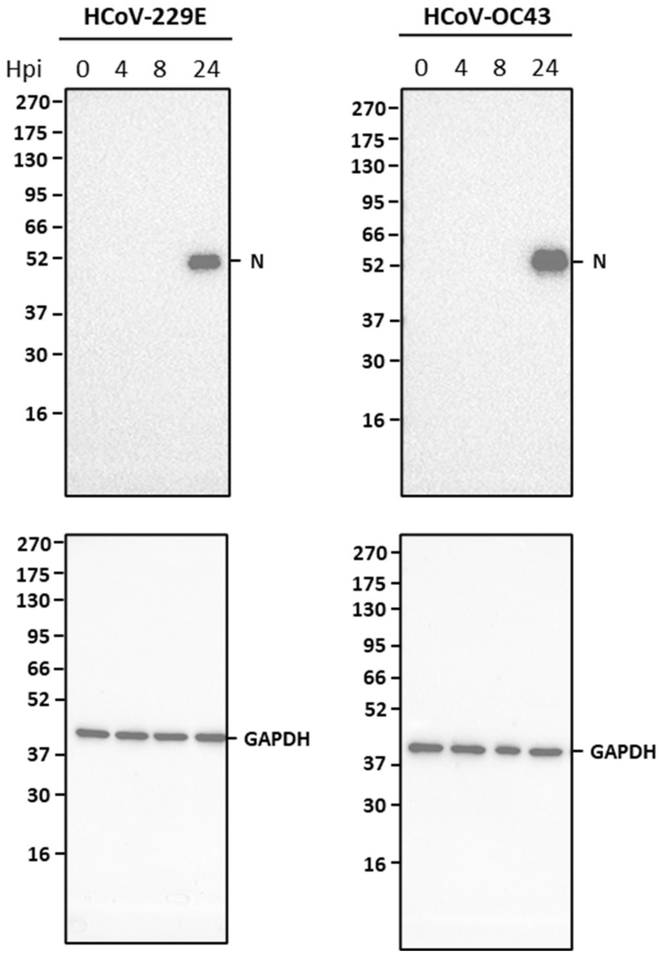


**Figure S5.** Full blot images of results shown in Figure 3B.

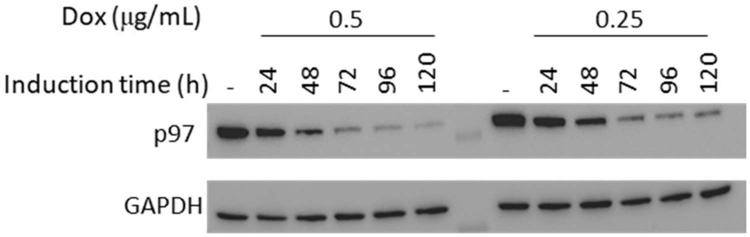


**Figure S6.** Knockdown of p97 expression in H1299 cells. H1299 cells were infected with lentivirus with Tet-regulated expression of p97-specific shRNA, or a control shRNA. Representative western blot images showing p97 expression and GAPDH (loading control) expression in cell lysates in the presence of Dox for different period.

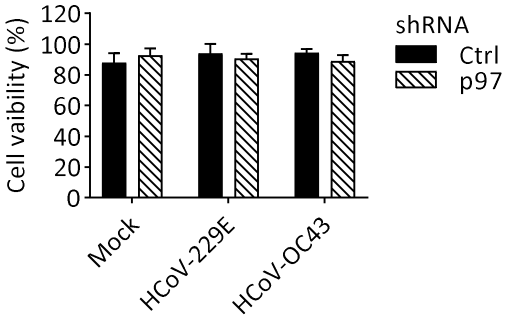


**Figure S7.** Cell viability of H1299 cells with inducible control shRNA (Ctrl shRNA) or p97 shRNA after HCoV infection. Cells were infected with HCoV-229E (MOI 0.05), HCoV-OC43 (MOI 0.01), or mock. After 1 h of infection, cells were washed and harvested at 0, 4, 8, or 24 hpi. Mock-infected cells were harvested at 24 hpi to quantify viral RNA level and viral N protein expression as shown in Figure 4A and Figure 4B. Cell viability of the harvested cells at 24 hpi was determined using Countess II Automated Cell counter. Error bars represent SD ($n$ = 3). No statistical significance was identified between groups using two-way ANOVA.

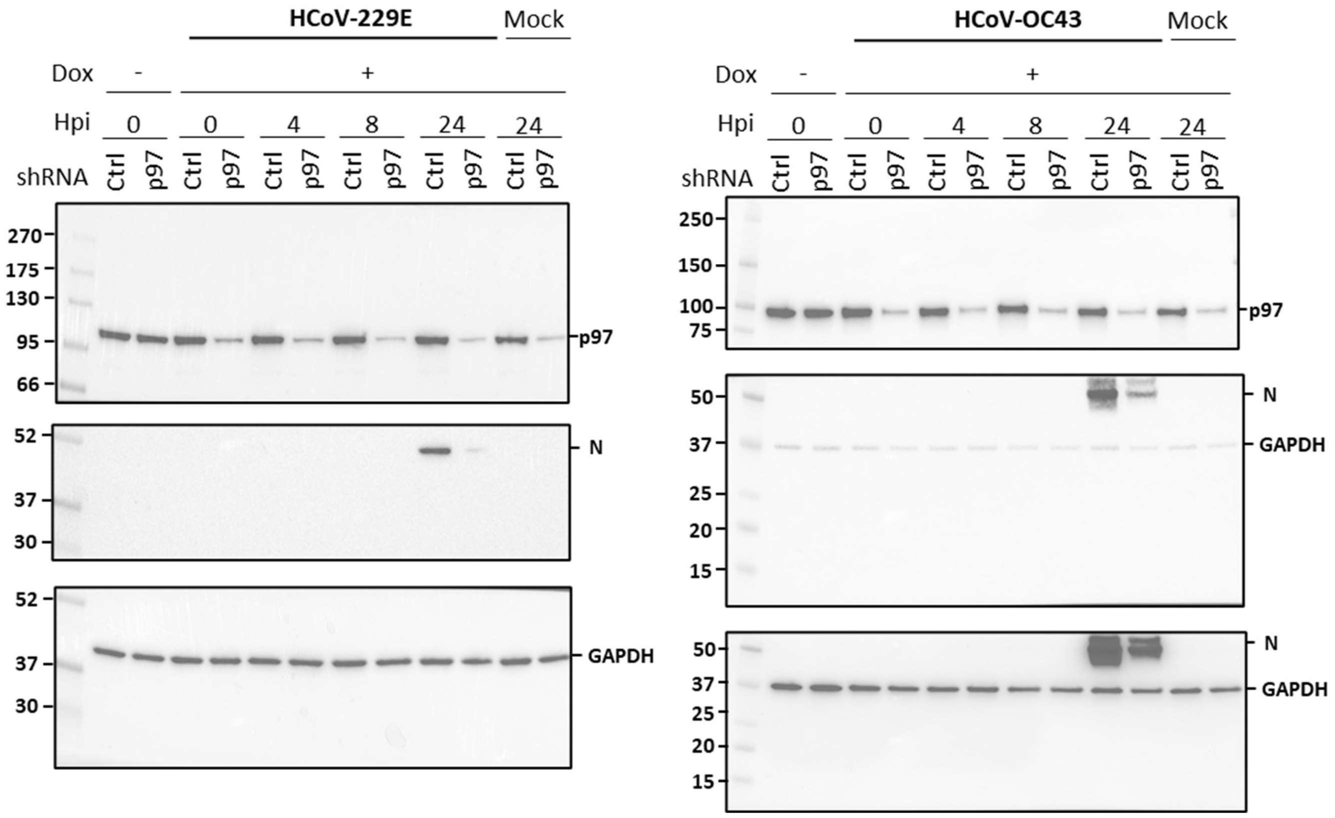


**Figure S8.** Full blot images of results shown in Figure 4B.

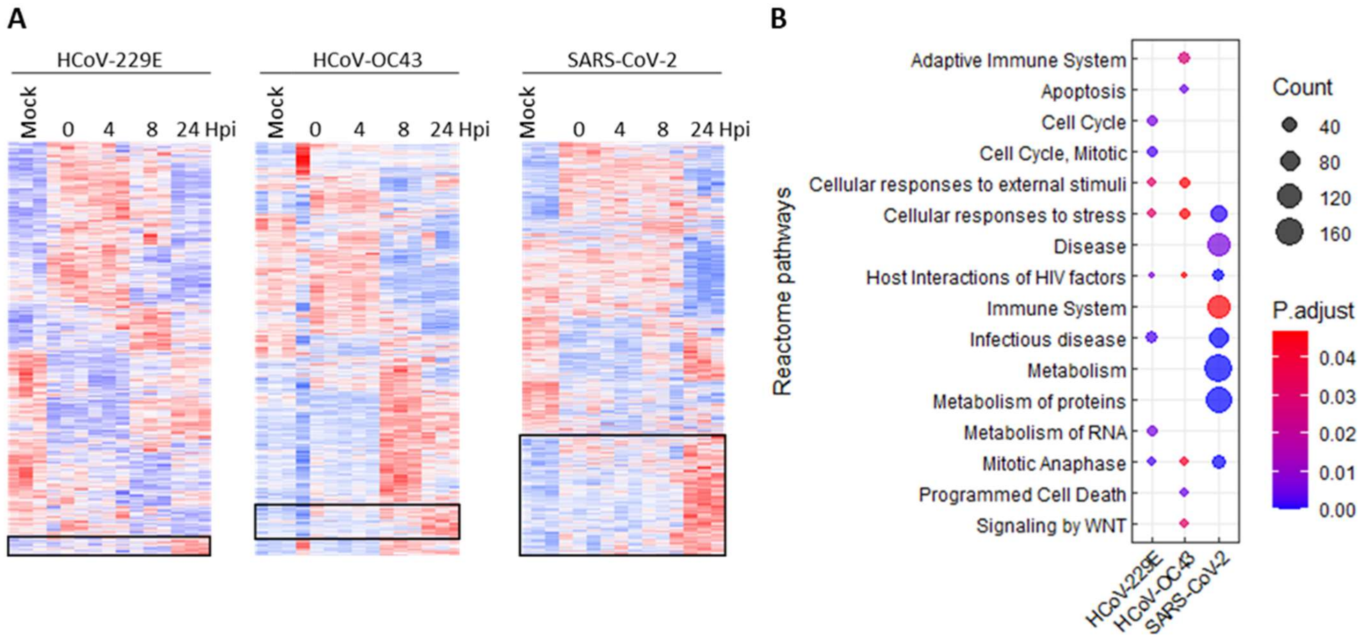


**Figure S9.** Overlapping host responses in cells after HCoV-229E, HCoV-OC43, or SARS-CoV-2 infection. (**A**) Protein levels change after HCoV-229E, HCoV-OC43, and SARS-CoV-2 infection. The proteins that were significantly up- or downregulated (Student's *t* test, $p < 0.05$) in the infected sample compared with the mock control are shown. Proteins that were increased over time after virus infection were selected for further analysis, including 109, 227, and 828 proteins in the HCoV-229E, HCoV-OC43, and SARS-CoV-2 cluster, respectively. Hierarchical cluster analysis was performed using Cluster 3.0. and TreeView. (**B**) Dotplot visualization of the top 5 and shared enriched Reactome pathway terms of proteins that were increased during the 24 h period after infection in each HCoV cohort (*p*-adjusted value < 0.05).

**A**

| Strains | Proteins in the Enriched Terms of "Cellular Responses to Stress" |
|---|---|
| HCoV-229E | MAPK7, PSMB5, RPL7, RPL23A, MAP2K4, TNIK, HSPA14, PSMA3, RPL35A, TUBB6, RPL6 |
| HCoV-OC43 | HSPA14, MRE11A, PSMD3, C11orf73, PSMB7, EZH2, MTOR, DCTN6, RELA, TUBB4B, DCTN1, PSMF1, TUBA1A, PSMD2, VCP |
| SARS-CoV-2 | PSME4, PSMA5, HSPA4L, CCAR2, PSMC3, PSMD13, DYNC1LI1, PSMD11, HSPA4, STIP1, PSMA1, PSME2, CAPZA2, MAPKAPK2, PSMD6, NBN, NUP54, TUBB4B, FAU, TUBB6, TUBA1C, HMGA1, P4HB, HSPA9, HSPA5, RPS28, RPLP2, RPL15, YWHAE, PSMB7, RPS4X, RPL35A, NUP210, RPLP0, NUP93, NUP160, RPS3A, ATP6V0C, TUBB3, DNAJA2, RPS8, RPL36, SOD2, TUBB2A, TUBB2B, ERO1A, EEF1A1, RPS12, PSMC5 |

**B**

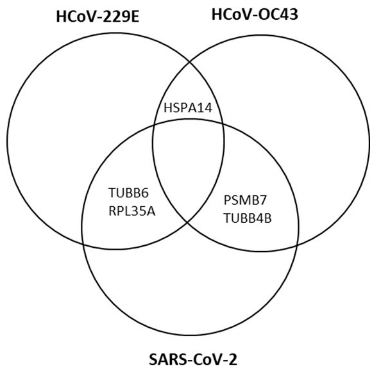


**C**

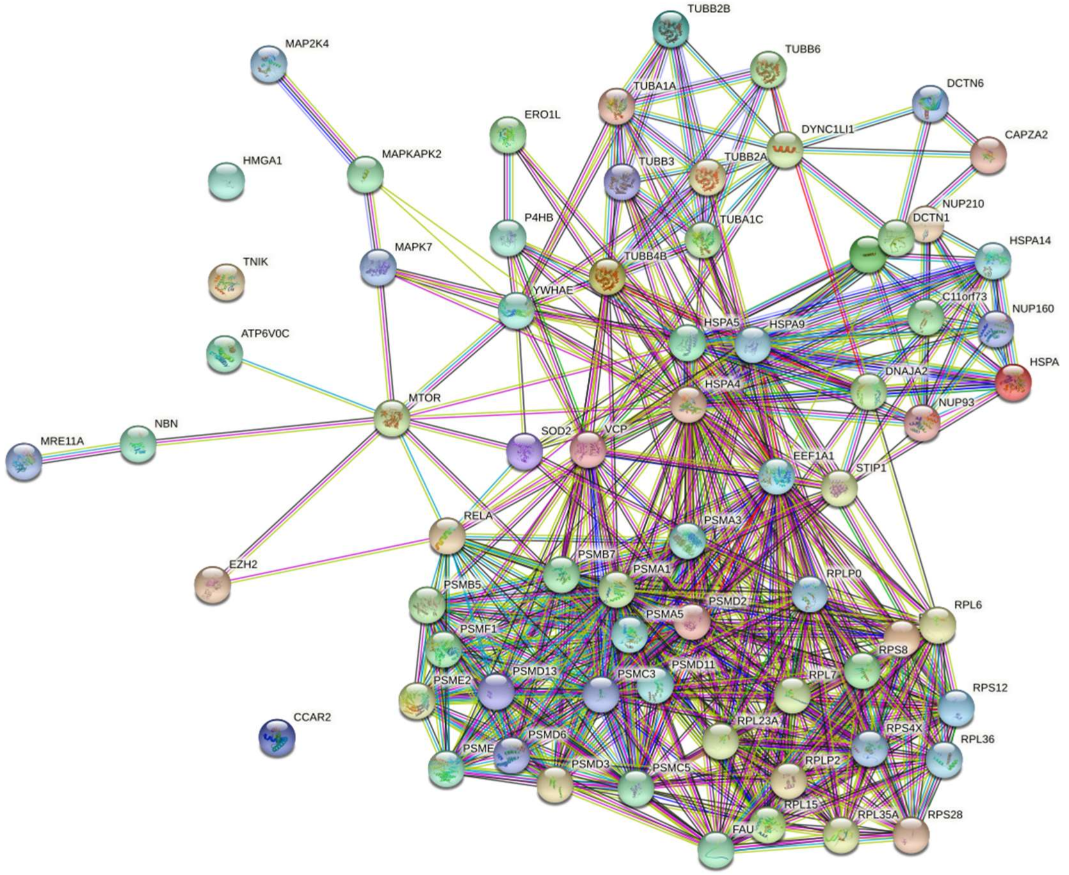

**Figure S10.** Proteins enriched in “Cellular responses to stress”. (**A**) List of proteins from three virus infection enriched in “Cellular responses to stress”. (**B**) Overlap analysis of proteins obtained from each infection. (**C**) Protein-Protein interaction network generated with STRING.

**A**

| **Strains** | **Proteins in the Enriched Terms of “Host Interactions of HIV Factors”** |
|---|---|
| HCoV-229E | PSMB5, KPNB1, AP2A1, AP1B1, XPO1, PSMA3 |
| HCoV-OC43 | AP1G1, PSMD3, PSMB7, PSMF1, PSMD2, RANGAP1 |
| SARS-CoV-2 | PSME4, PSMA5, RCC1, PSMC3, PSMD13, AP2A1, PSMD11, PSMA1, PSME2, PSMD6, RAN, NUP54, AP1M1, HMGA1, NPM1, SLC25A5, RANGAP1, PSMB7, NUP210, NUP93, NUP160, RAC1, PSMC5 |

**B**

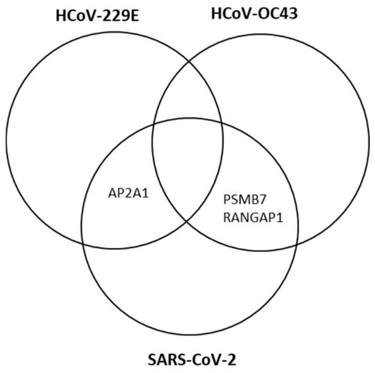


C

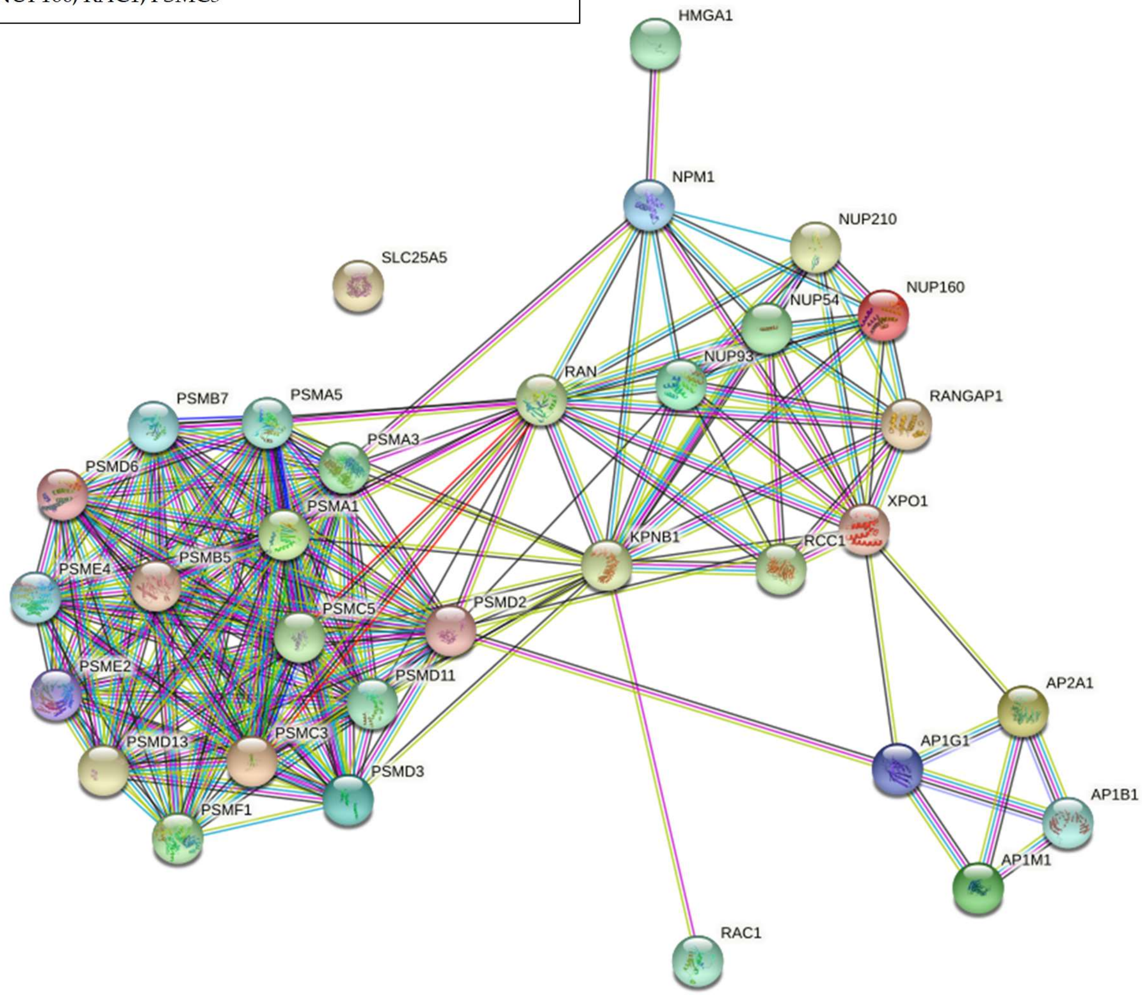


**Figure S11.** Proteins enriched “Host interactions of HIV factors”. (**A**) List of proteins from three virus infection enriched in “Host interactions of HIV factors”. (**B**) Overlap analysis of proteins obtained from each infection. (**C**) Protein-Protein interaction network generated with STRING.

**A**

| Strains | Proteins in the Enriched Terms of "Mitotic Anaphases" |
|---|---|
| HCoV-229E | SIRT2, PSMB5, CHMP4A, STAG1, KPNB1, ERCC6L, XPO1, PSMA3, TUBB6 |
| HCoV-OC43 | PSMD3, PSMB7, TUBB4B, KNTC1, PSMF1, PPP2R5C, TUBA1A, PSMD2, RANGAP1 |
| SARS-CoV-2 | PSME4, TNPO1, UBE2I, PSMA5, PPP2R5C, RCC1, PSMC3, PSMD13, DYNC1LI1, PSMD11, ZWILCH, PSMA1, PSME2, PSMD6, RAN, NUP54, TUBB4B, TUBB6, TUBA1C, RANGAP1, PSMB7, STAG1, NUP93, NUP160, TUBB3, TUBB2A, TUBB2B, PSMC5 |

**B**

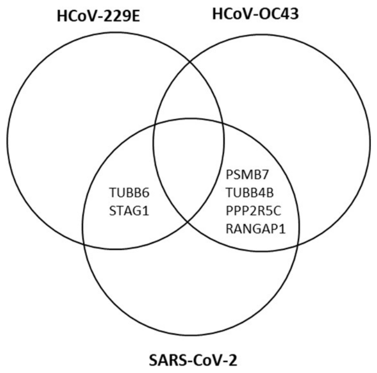


**C**

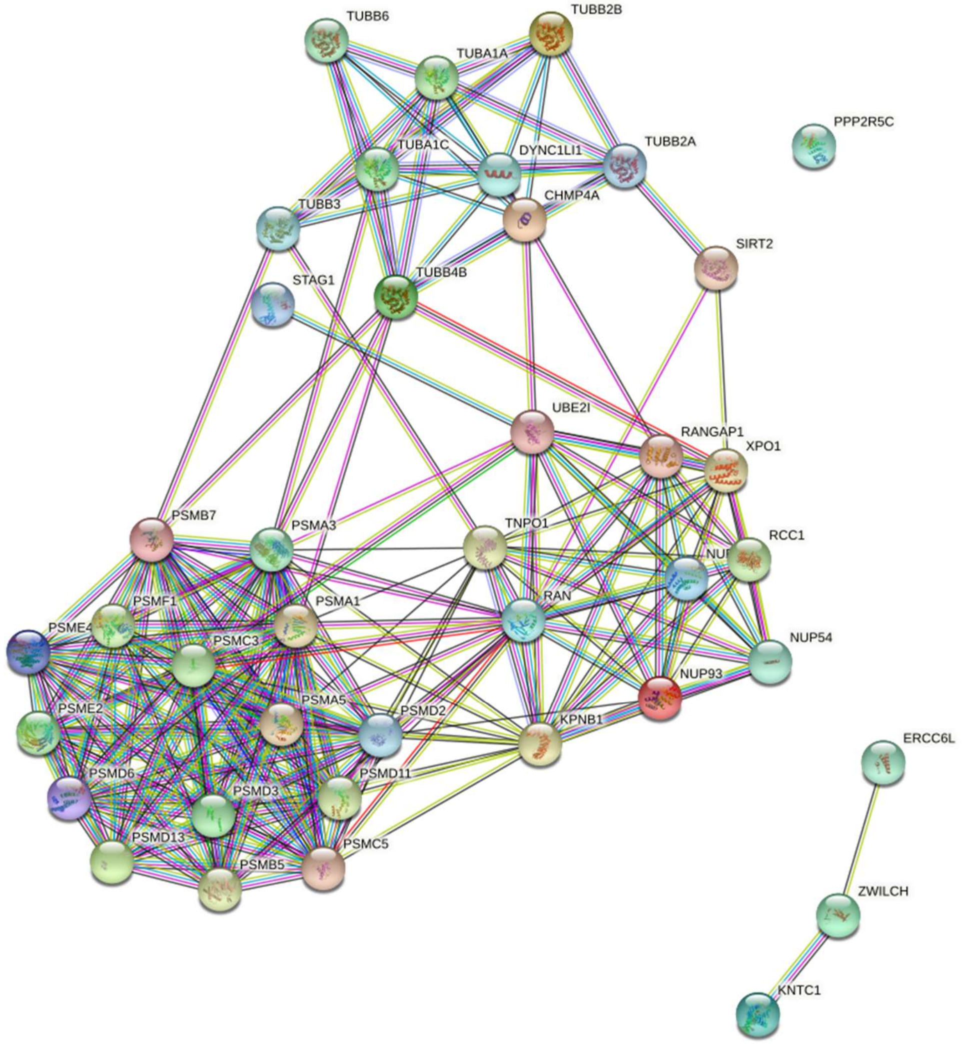

**Figure S12.** Proteins enriched "Mitotic anaphases". **(A)** List of proteins from three virus infection enriched in "Mitotic anaphases". (**B**) Overlap analysis of proteins obtained from each infection. (**C**) Protein-Protein interaction network generated with STRING.

**Table S1**. TagMan probes list

| | **Assay ID** | **Gene Symbol** | **Entrez Gene ID** |
|---|---|---|---|
| **HCoV-229E** | Vi06439671_s1 | Human coronavirus 229E | Human coronavirus 229E |
| **HCoV-OC43** | Vi06439646_s1 | Betacoronavirus 1 | Betacoronavirus 1 |
| **GAPDH** | Hs02786624_g1 | GAPDH | 2597 |

**Table S2.** Proteomics of H1299 cells with control shRNA and p97 shRNA after HCoV-229E.

**Table S3.** Proteomics of H1299 cells with control shRNA and p97 shRNA after HCoV-OC43

**Table S4.** Reactome pathway enrichment analysis

**Table S5.** Multi-list Reactome pathway enrichment analysis
